# Enhancing Sorghum Yield Through Efficient Use of Nitrogen – Challenges and Opportunities

**DOI:** 10.3389/fpls.2022.845443

**Published:** 2022-02-28

**Authors:** Troy J. Ostmeyer, Rajeev Nayan Bahuguna, M. B. Kirkham, Scott Bean, S. V. Krishna Jagadish

**Affiliations:** ^1^Department of Agronomy, Kansas State University, Manhattan, KS, United States; ^2^Center for Advanced Studies on Climate Change, Dr. Rajendra Prasad Central Agricultural University, Samastipur, India; ^3^Grain Quality and Structure Research Unit, CGAHR, USDA-ARS, Manhattan, KS, United States

**Keywords:** grain quality, high-throughput phenotyping, sensors, stay-green, source-sink relationships

## Abstract

Sorghum is an important crop, which is widely used as food, forage, fodder and biofuel. Despite its natural adaption to resource-poor and stressful environments, increasing yield potential of sorghum under more favorable conditions holds promise. Nitrogen is the most important nutrient for crops, having a dynamic impact on all growth, yield, and grain-quality-determining processes. Thus, increasing nitrogen use efficiency (NUE) in sorghum would provide opportunities to achieve higher yield and better-quality grain. NUE is a complex trait, which is regulated by several genes. Hence, exploring genetic diversity for NUE can help to develop molecular markers associated with NUE, which can be utilized to develop high NUE sorghum genotypes with greater yield potential. Research on improving NUE in sorghum suggests that, under water-deficit conditions, traits such as stay-green and altered canopy architecture, and under favorable conditions, traits such as an optimized stay-green and senescence ratio and efficient N translocation to grain, are potential breeding targets to develop high NUE sorghum genotypes. Hence, under a wide range of environments, sorghum breeding programs will need to reconsider strategies and develop breeding programs based on environment-specific trait(s) for better adaptation and improvement in productivity and grain quality. Unprecedented progress in sensor-based technology and artificial intelligence in high-throughput phenotyping has provided new horizons to explore complex traits *in situ*, such as NUE. A better understanding of the genetics and molecular pathways involving NUE, accompanied by targeted high-throughput sensor-based indices, is critical for identifying lines or developing management practices to enhance NUE in sorghum.

## Introduction

Sorghum [*Sorghum bicolor* (L.) Moench, Poaceae] is an important C_4_ crop that is mainly utilized for human food, animal feed, forage, and fodder, but it is also an important source of fiber and feedstock for biofuel production (Bollam et al., 2021). Sorghum is grown worldwide and distributed across different continents including North America, Africa, Asia, and Australia (Kimber, 2000; Morris et al., 2013). Sorghum is a staple food for the majority of the population in semi-arid tropical regions of Africa and Asia (Buffo et al., 1998; Dicko et al., 2006). Hence, improving the resource-use efficiency, yield, and quality in grain sorghum would affect large number of people around the world. In general, sorghum is known to have adapted to marginal soils with poor nutrient supply (Qi et al., 2016) and is tolerant to abiotic stresses, such as heat and drought (Pennisi, 2009; Sunoj et al., 2017; Chiluwal et al., 2018), allowing it to be productive even under unfavorable and marginal environments (van Oosterom et al., 2001; Saballos, 2008). However, sorghum yields may increase substantially with an adequate supply of nutrients. For example, higher grain yields under intensive agricultural systems have been achieved with adequate nitrogen (N) supply (Mahama et al., 2014; Bollam et al., 2021). Research on sorghum-yield improvement has been focused mainly on gaining smaller increments under marginal and unfavorable environments, and limited efforts have been invested in exploiting the true genetic potential of sorghum. Nevertheless, understanding the source-sink dynamics and the ability to enhance nitrogen use efficiency (NUE) in sorghum, including the traits or mechanisms involved in N uptake, transport, and remobilization, could provide new opportunities to achieve significant genetic gain under relatively favorable environments.

Nitrogen is an important essential nutrient and the most limiting one in modern crop production (Hirel et al., 2011). It plays an important role in the production of amino acids, proteins, and pigments, e.g., chlorophyll (Dovale et al., 2012). Depending on soil properties, plants absorb two chemical forms of N from the soil, viz., nitrate (NO_3_^–^) and ammonium (NH_4_^+^) (Barber, 1984; Crawford and Glass, 1998). However, in well-aerated soils, NO_3_^–^ is the most abundant and available form of N for plants (Warncke and Barber, 1973), and NH_4_^+^ tends to be the predominant form in non-cultivated soils (Crawford and Glass, 1998). Conversely, when both forms of N are available, NO_3_^–^ tends to be the preferred form absorbed by plants (Pathak et al., 2008).

Adequate soil moisture is an important factor determining the efficient utilization of N by plants (Silva and Uchida, 2000), because low N availability in water-deficient soils substantially reduces crop yields. Hence, in drought-prone and rain-fed agricultural systems, effective utilization of applied N fertilizers is a major challenge in alleviating N deficiencies in crops, including sorghum (Lemaire et al., 1996; Andrews et al., 2013; McHenry, 2016). Besides soil moisture, temperature and pH are documented as influencing N-absorption rate in crops (Lycklama, 1963; Warncke and Barber, 1973).

Commercial sources of N fertilizers can be solubilized easily, which makes N readily available for absorption by plants. Hence, these fertilizers are most widely used in modern agriculture (Hirel et al., 2011). In the last 40 years, use of commercial fertilizers has increased > 7-fold, which has doubled crop yields. But it has resulted in low fertilizer use efficiency, particularly low NUE in crops (Tilman et al., 2002; Hirel et al., 2011). A major reason for low NUE in crops is due to an estimated 50–55% of nitrogen lost by leaching of nitrate, nitrous oxide emission, ammonia volatilization, and poor N uptake in water-deficient soil (Raun and Johnson, 1999; Ma et al., 2019; *reviewed in* Mahmud et al., 2021). Further, efficiency of multiple processes involved in the N loss from the soil is variable with environment and soil type (Mahmud et al., 2021). Incorporating NUE traits in crop-yield-improvement programs is crucial to reduce the overuse of commercial nitrogen fertilizers, associated cost, and nitrous oxide (N_2_O) emissions into the environment (Good et al., 2004; Ahlgren et al., 2008; Dovale et al., 2012).

Exploring natural, genetic diversity for NUE is one of the potential strategies that can be used to enhance NUE in sorghum. This would involve integration of high-throughput phenotyping (HTP), next-generation-sequencing (NGS)-based genotyping technologies, -omics analysis, and identification of candidate gene(s) (Masclaux-Daubresse et al., 2010; Bollam et al., 2021). However, physiological traits and molecular pathways that affect N uptake, assimilation, and remobilization in plants are complex (Gastal and Lemaire, 2002), and they get further complicated when interactions of N metabolism with different environmental factors are considered (Nasraoui et al., 2013; Das et al., 2017). Breeding for hybrids in soils that contain high available N may have already resulted in an indirect selection of sorghum hybrids with high NUE (Ceccarelli, 1996). However, for trait-based, targeted breeding aimed at increasing NUE, understanding the complexity and identifying key traits that will allow for increased NUE are essential to develop novel and highly efficient sorghum hybrids.

This review aims to highlight and summarize the importance of enhancing NUE in sorghum and to discuss the role of conventional, as well as advanced high-throughput phenotyping, in determining genetic diversity for NUE in sorghum. The major objectives are to (i) ascertain the factors that impact N status and eventually NUE in grain sorghum, (ii) identify key physiological traits and key genes that can help improve NUE and increase grain yield and quality in sorghum, and (iii) explore the role of high-throughput, sensor-based phenotyping to strengthen breeding programs for developing nitrogen-use-efficient sorghum hybrids. Finally, future research directions for increasing NUE in sorghum and developing hybrids with high yields and improved grain quality are proposed.

## Impact of Nitrogen Levels on Biomass, Grain Yield, and Grain Nutrients in Sorghum

Sorghum is commonly grown in soils with low fertility and water availability, which drives the need for efficient use of resources, including N (van Oosterom et al., 2001). Extensive research has been conducted on the impact of available N on final grain yield. But studies on grain quality are limited (Kaufman et al., 2013; Mahama et al., 2014; Melaku et al., 2017). Key grain-yield determining components of sorghum are grain number and weight. The number of kernels is determined during the early part of the growth cycle (panicle initiation, gametogenesis, and anthesis), while kernel weight is mainly driven by post-flowering processes (Maman et al., 2004). Mahama et al. (2014) found that sorghum grain yield increased with an increase in applied N (0, 45, and 90 kg N ha^–1^), which was attributed to an increase in grain number. Worland et al. (2017) reported that kernel size in sorghum could be manipulated by N-application timing, because increased N supply at the grain-filling stage could increase sink size (kernel size), which would eventually support further increase in grain yield. Nevertheless, kernel number is considered as the major factor driving the overall yield of sorghum. Thus, application of N at key developmental stages, such as panicle initiation and spikelet differentiation, is crucial to achieve higher yields. Further, it has been documented that optimum N levels could aid in active and extended periods of photosynthesis, which result in enhanced biomass and grain yield in sorghum (Zhao et al., 2005). Interestingly, the effect of water stress on yield components has been shown to be similar to nitrogen deficiency. It has been documented that maintaining grain number, even under post-flowering water deficit, was more important than maintaining individual grain weight in sorghum (Adotey et al., 2021).

**FIGURE 1:**
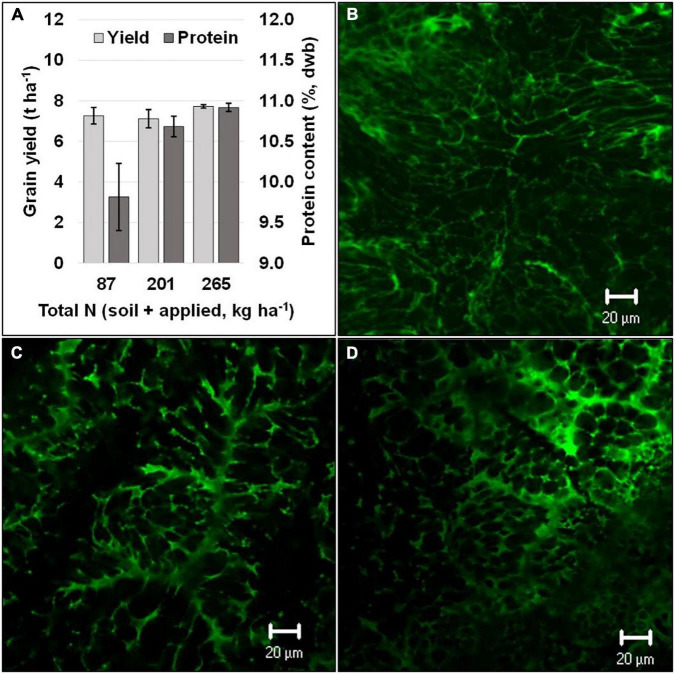
Grain yield and protein (dry weight basis) at different N levels **(A)** and a visual comparison of confocal laser scanning micrographs of dried grain endosperm (green color obtained using fast green dye indicates protein) with differing grain protein content respective to the total N levels [**(B)** 87 kg ha^−1^, **(C)** 201 kg ha^−1^, and **(D)** 265 kg ha^−1^]. Black vertical bars overlaying the gray bars **(A)** represent standard error (*n* = 4).

In addition to grain yield and biomass, several studies have confirmed that there is an increase in grain-protein content in sorghum with an increase in available N (Bayu et al., 2006; Kaye et al., 2007; Rashid et al., 2008; Kaufman et al., 2013). Several studies showed a positive linear response between N-application rate and grain-protein content (Bayu et al., 2006; Rashid et al., 2008; Diallo, 2012). Moreover, split applications of N at panicle initiation and at booting revealed that the optimum level of N application for yield and grain-protein content could vary (Figure 1; Ostmeyer et al., UnPub). Despite a non-significant change in yield under differential levels of N, protein levels increased substantially, which suggests that opportunities exist to enhance grain protein in sorghum independent of yield (Figure 1). On the contrary, few studies have reported the impact of N-application rates on starch content in sorghum grains (Kaufman et al., 2013). Although trade-offs between carbon and nitrogen assimilation have been documented (Tateno and Chapin, 1997), it has been reported that starch content and granule-size distributions were not affected by varying N-application rates (Kaufman et al., 2013). Similarly, the effect of N application on other grain macro- and micro-nutrients in sorghum has received little attention. One study reported that N application did not alter mineral concentrations in sorghum grains, except for P and S contents, and the results showed that there was a higher P content and a lower S content with increasing N application (Kaufman et al., 2013). Future research should focus on the ability to enhance grain protein and changes in amino-acid composition. In addition, concentration of macro- and micro-nutrients in sorghum grain, based on the quantity and timing of N application, warrant attention.

## Nitrogen Partitioning and Stay-Green Trait in Sorghum

When sorghum is grown under N-sufficient conditions, shoots have been shown to accumulate higher concentrations of N than roots (Gleadow et al., 2016). Moreover, it has been shown that, until anthesis, the stem accumulates a greater amount of N than the leaves, whereas the panicle accumulates the majority of the plant’s N during later grain-filling stages due to remobilization of accumulated N from leaves and stem (McHenry, 2016). This partitioning of N within sorghum plant parts has been documented to follow the same trend with low- and high-input treatments, as well as under rain-fed or irrigated conditions (McHenry, 2016). Contrary to these results, it has been reported that leaf sheaths and leaf blades are the predominant N sink in sorghum, and high levels of N application (10 mM KNO_3_) given at 55 days after sowing resulted in a fourfold higher nitrate concentration in leaf sheaths compared to the control (Worland et al., 2017). Moreover, under N-deficient conditions, the demand for N from the leaves becomes proportionally greater than that from the stem (van Oosterom et al., 2010a). The higher accumulation of N in the leaves helps to maintain higher rates of photosynthesis, which are linked to increased pigment (chlorophyll) levels (Muchow and Sinclair, 1994). In the later part of the growth cycle, remobilization of N to panicles increases panicle N concentration during anthesis. Nitrogen concentrations in sorghum reach their highest levels during the grain-filling stage (Worland et al., 2017). van Oosterom et al. (2010b) reported that the first half of grain filling in sorghum is sink limited and the available N amount has no impact on N accumulation within the grain. However, during the second half of the grain-filling stage, N accumulation can be affected by the available N amount, leaving the plant source limited. This finding is further supported by confocal laser scanning micrographs and analysis of protein content (dry weight basis), as presented in Figure 1 [for additional details on scanning see Impa et al. (2020)]. There are distinctly lower protein concentrations at lower N levels.

The stay-green trait is a phenomenon that results in delayed foliar senescence, particularly during terminal grain filling. It aids in extended duration of assimilate production (functional stay-green), which ultimately results in higher yield in many crops (Thomas and Ougham, 2014). The stay-green phenomenon has been shown to be highly relevant under stressful conditions such as drought stress (Jordan et al., 2012; Thomas and Ougham, 2014). A negative correlation between the sorghum grain size and relative rate of leaf senescence (*r* = −0.63), and a positive correlation between grain yield and green-leaf area (*r* = 0.64), were observed under terminal drought (Borrell et al., 1999). The stay-green trait in sorghum has been reported to be affected by soil N availability and remobilization of N from vegetative parts to developing grains (Addy et al., 2010). In a controlled-environment study, Addy et al. (2010) showed that the stay-green trait in sorghum can only be expressed when an adequate amount of N is available in the soil. Findings have suggested that the application of N prior to the reproductive stage is necessary to increase the chlorophyll content at anthesis and the grain-filling stage, which favors higher grain yields under favorable conditions. On the contrary, under severe water-deficit stress, by reducing canopy size and shifting water use from pre- to post-flowering, more water is retained in the soil and photosynthetic processes are extended (Borrell et al., 2014). Under these dry conditions, altered canopy architecture was observed with reduced tillering, changes in leaf anatomy such as decreased leaf size, especially at the top of the plant, and reduced number of leaves per culm (Borrell et al., 2014). In summary, under favorable conditions, the stay-green trait is retained by having continued N availability, and, under water-deficit stress, altered canopy and leaf anatomy retain the stay-green trait. Thus, the stay-green trait sustains productivity under a wide range of environments with varying moisture conditions.

Nitrogen is an essential element for maintaining the chlorophyll content in plants, which is necessary for photosynthetic activity (Dovale et al., 2012). Thus, higher N demand by stay-green hybrids during the grain-filling period can be met by a higher N uptake from soil. In contrast, senescing phenotypes (those that do not have the stay-green trait) need a larger proportion of the grain N to be supplied through remobilization from stem and leaves (Borrell and Hammer, 2000). The stay-green trait has been shown to be effective in minimizing impacts of drought stress. Under greenhouse conditions, Addy et al. (2010) showed that the expression of the stay-green phenotype in sorghum was contingent upon the concentration of N, and plants needed at least 1.35 g N plant^–1^.

Limited knowledge is available on the impact of variable N availability on the stay-green trait in sorghum, and, hence, interactions of stay green and N warrant further investigation. Demonstration of the effectiveness of the stay-green trait under harsh environmental conditions (Harris et al., 2007; Borrell et al., 2014) has resulted in routine incorporation of the trait in almost all sorghum hybrid-breeding programs around the world (Jordan et al., 2012). Under sufficient resource (water and nutrients) availability, studies have shown that sorghum can yield as much as 9.3 t ha^–1^ (Assefa and Staggenborg, 2010) and 10.5 t ha^–1^ (Jordan et al., 2012). These studies showing the maximum yield of sorghum demonstrate the opportunity to enhance productivity and overall NUE under low-to-moderate-stress environments.

**FIGURE 2:**
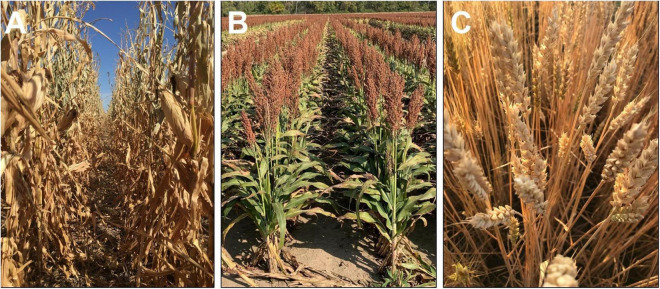
Visual comparison of plant greenness of maize (**A**; Photo credit to Rachel Veenstra), grain sorghum **(B)**, and winter wheat **(C)** after physiological maturity and close to harvest maturity. Each image was captured when the respective crop was ready for mechanical harvesting.

Interestingly, other cereals, such as wheat cultivars or maize hybrids, have either a complete or a major proportion of senescence achieved after reaching physiological maturity, while sorghum with the stay green trait continues to retain much of its green foliage even at harvest (Figure 2). This poses the question, “If the blanket inclusion of the stay-green trait is used in all breeding programs (Jordan et al., 2012), does this lead to a disadvantage under less stressful or non-stress conditions?” The hypothesis behind the stay-green phenomenon is that the carbon (sugars in the green stems) and N in the green leaves (Figure 2B) are locked in the shoot biomass, which has the potential to further enhance yield or grain protein content through increased translocation. Recent findings using lysimeter and field-based rainout shelters indicate the lack of reduction in individual grain weight at different positions along the panicle, even under severe drought (Adotey et al., 2021). Thus, as a route to enhance productivity under less stressful environments, there is potential to utilize these resources locked in the stem and leaves to support a larger sink size in sorghum. Moreover, a careful consideration of N translocation due to onset of senescence during post-flowering phase is warranted to fine-tune the balance between stay-green and senescence to achieve optimum grain yield and quality [reviewed in Jagadish et al. (2015)].

## Physiological and Molecular Approaches to Enhance Nitrogen Use Efficiency in Sorghum

### Physiological Approaches

Nitrogen uptake in the form of nitrate and ammonium from the soil and its assimilation in plants are crucial to increase NUE (Xu et al., 2012). While nitrogen uptake is regulated by soil physical properties (Shamme et al., 2016), symbiotic microbial associations (Courty et al., 2014), and root traits, assimilation of N is more complex and involves different enzymes and associated co-factors (Massel et al., 2016). Pathak et al. (2008) summarized key physiological aspects of N uptake and assimilation, which can contribute to high NUE in plants such as (i) optimized nitrate uptake from the rhizosphere; (ii) increased nitrate and nitrite reduction efficiency (crucial enzymes to convert nitrate to nitrite and nitrite to ammonium for N assimilation), and (iii) increased ammonium assimilation. Bhatt et al. (1979) reported that a decrease in plant height was associated with an increase in nitrate reductase activity in sorghum. Higher nitrate reductase activity contributing to an augmented rate of N assimilation eventually resulted in higher NUE in shorter plants. However, limited information is available on the physiological or morphological traits that can be used to identify high NUE phenotypes for large-scale screening of sorghum germplasm. Conversely, a significant (*p* ≤ 0.01), positive relationship was observed between total leaf N and CO_2_ assimilation and the activity of the enzymes involved in carboxylation, including Rubisco and phosphoenolpyruvate carboxylase in C_3_ (*r* = 0.81) and C_4_ (*r* = 0.77) crops, respectively (Sage et al., 1987). Under N-deficit conditions, Rubisco is preferentially synthesized and can account for 50% of the total N in maize seedlings (Sugiyama et al., 1984).

### Candidate Gene-Based Approaches to Enhance Nitrogen Use Efficiency

Improving NUE in crops has been attempted through a transcription-factor approach, wherein the *Zea mays* DOF1 (ZMDof1) transcription factor was introduced into sorghum, targeting phosphoenolpyruvate carboxylase (Peña et al., 2017). A tissue-specific maize rbcS1 promoter and a constitutive UBI4 promoter from sugarcane with the same ZMDof1 were used to increase biomass and yield components and down-regulate genes involved in photosynthesis that had negative impacts on height and biomass, respectively (Peña et al., 2017). Despite the positive agronomic impact achieved by following a tissue specific transcription-factor approach, a more systematic transgene stacking was considered essential to enhance NUE in crops, with minimal trade-offs under natural field conditions. A number of studies have overexpressed key N assimilation enzymes, including nitrate and nitrite reductase and glutamine synthetase (ammonium assimilation), but they have produced inconsistent results in enhancing NUE in cereal crops, such as rice (Massel et al., 2016, and references within). Several genes have been identified in sorghum (mostly *nitrate/peptide transporter 1* (*NRT1/PTR*) genes contributing to N uptake), which show a high genetic diversity and could be utilized for further enhancement of NUE (Massel et al., 2016). Though a number of studies have manipulated different genes in different plant species, a dominant gene that enhances NUE has not been identified, which demonstrates the complexity surrounding the enhancement of NUE (Pathak et al., 2008). Further research is needed to ascertain how NUE is affected by photosynthetic rate, respiration rate, and other physiological factors controlled at the genetic level and altered by gene and environmental interactions.

## Sensor-Based High-Throughput Phenotyping for Determining Nitrogen Use Efficiency

Responses of crops to nitrogen is mainly determined by their genetic background, and the complexity of NUE-related traits is controlled by multiple genes (Bollam et al., 2021). Thus, exploring genetic diversity for key traits associated with NUE is a promising route to develop high-NUE sorghum hybrids. Moreover, use of high-throughput phenotyping would allow for screening large and diverse germplasm or populations in a short period of time, which would facilitate the inclusion of NUE-related traits into routine breeding programs. High-throughput, sensor-based phenotyping is increasingly being used to monitor N status of crop plants and to devise appropriate N management tools (Yang et al., 2009; Shafian et al., 2018). Use of unmanned aerial vehicles (UAVs), equipped with visible light and multispectral sensors, has become popular recently (Xue and Su, 2017). These sensor-based platforms operate by measuring light absorption, transmittance, and reflectance. They rely on the principle that known wavelengths of light are absorbed by chlorophyll and are used for energy in photosynthesis to support growth and productivity. The part of light that is not absorbed by the plant is either transmitted or reflected, and different sensors or cameras are equipped to quantify transmittance and reflectance values. These values can be combined into an index that is used to compare traits that are strongly correlated with tissue nitrogen content, water content, chlorophyll content, and other pigments in sorghum under different management practices (Shafian et al., 2018).

Different spectral regions of electromagnetic radiation (EMR), such as the visible and near infrared (NIR), can be used in algorithms called vegetation indices (VIs) to determine different biological properties in sorghum (Shafian et al., 2018). A common vegetative index is the NDVI (normalized difference vegetation index), which is obtained from the ratio between the red and NIR reflectance. The sensors and cameras for sensing and imaging have become highly efficient, reliable, and precise for estimating crop health and greenness, which indirectly indicates N status in crops including sorghum (Fiorani and Schurr, 2013). Spectral image analysis has been used for the quantification of sorghum growth and grain yield. For instance, Yamamoto et al. (2002) found that the SPAD value of sorghum leaves was related to the chlorophyll level. Similarly, NDVI values of early growth stages could be used to estimate plant density (Potgieter et al., 2017; Shafian et al., 2018) as well final grain yield (Shafian et al., 2018). Moges et al. (2007) showed that NDVI data collected at panicle initiation in sorghum was highly correlated with final grain yield. A study observing the senescence of multiple grain sorghum hybrids with differing levels of stay-green showed that, when data were collected at anthesis, the NIR band (750–950 nm) alone related best to biomass, while the green-NDVI index related best to final grain yield (Barnhart et al., 2021).

Using modeling techniques, functional relationships between leaf reflectance and leaf N concentrations have been established (Zhao et al., 2005). Zhao et al. (2005) showed that the visible regions of the EMR are highly sensitive to N concentration. They determined that the leaf reflectance at 555 (blue) and 715 nm (red) increased when N was deficient and were related to either N or chlorophyll concentrations. Zhao et al. (2005) also reported that specific reflectance ratios (1,075 nm/735 nm and 405 nm/715 nm) could be used in the estimation of leaf N and chlorophyll concentrations with high accuracy. Using an UAV, Li et al. (2018) found that the chlorophyll and N content were correlated to green chlorophyll, red-edge chlorophyll, and normalized difference red-edge (NDRE) indices, as well as measurements taken with a ground-based hyperspectral sensor that was clipped to a sorghum leaf. They showed that UAV technology has the potential to be useful in phenotyping sorghum for key NUE traits, including biomass, chlorophyll, and nitrogen contents.

Similar to the determination of greenness or the overall health of crops, ground- or aerial-based sensors allow for efficient detection of the start of post-flowering senescence and provide opportunities for developing optimized source-sink relationships (Potgieter et al., 2017; Šebela et al., 2020; Barnhart et al., 2021). Barnhart et al. (2021) demonstrated that red-green-blue (RGB) VIs, specifically the visible atmospherically resistant index (VARI), have the potential to detect sorghum senescence rates (*r* = 0.60). Using NDRE, Potgieter et al. (2017) were able to observe the rate of senescence (difference between NDRE values at maximum canopy cover and at harvest maturity) of known stay-green and senescent sorghum genotypes, and found that the senescent genotypes had a faster rate of senescence as compared to the stay-green genotypes. Identifying the duration of leaf greenness (extended under water-deficit conditions; see section “Breeding for Enhanced Nitrogen Use Efficiency in Sorghum”) can be implemented using sensor-based technology. The development of more user-friendly image collection and processing procedures, along with the decrease in the cost of technology, is allowing more producers and researchers to take advantage of photogrammetry technologies (Barnhart et al., 2021). The use of these technologies has also the potential to increase the accuracy and speed of breeding for enhanced NUE, yield, and other key traits in sorghum.

Integration of these high-throughput phenotyping tools with advanced next generation sequencing-based genotyping would help to explore potential genetic variability for nitrogen utilization efficiency in sorghum. A native genetic variability in key yield traits related to NUE has been reported in a diverse set of sixty sorghum genotypes under low nitrogen regime (Bollam et al., 2021). Thus, potential genetic variability for N uptake and utilization efficiency exists and can be exploited by evaluating larger sets of diverse genotypes with high-throughput phenotyping and genotyping tools. Moreover, sensor-based N application along with integrated agronomic practices such as supplemental irrigation (Sigua et al., 2018), planting density (Dembele et al., 2021) could achieve higher yield and NUE in sorghum. Conversely, a higher genetic diversity has been reported in sorghum for below ground traits as compared to the aboveground traits (Wojciechowski and Kant, 2021). Roots architecture is crucial for uptake and supply N and other nutrients. Indeed, root traits such as root length, root tips, root average diameter, root weight, and specific root length were crucial determinants for NUE under low N conditions in wheat and spinach (Awika et al., 2021; Puccio et al., 2021). However, information on different root trait combinations related to NUE in sorghum warrants further research.

## Breeding for Enhanced Nitrogen Use Efficiency in Sorghum

Breeding programs and genetic-modification studies have made modest progress in enhancing NUE in cereal crops, including sorghum, due to the complex nature of the trait and the lack of accurate phenotyping protocols for NUE (Garnett et al., 2015). Additionally, yield-oriented breeding programs have utilized narrow genetic diversity for N use (Massel et al., 2016) and NUE (Han et al., 2015). Grain yield is the major trait that is used extensively in determining NUE, which further complicates efforts to enhance NUE, because grain yield is influenced by the prevailing environmental conditions and strong genotype by environment interactions (Nguyen and Kant, 2018). Despite high N utilization efficiency (biomass produced in relation to N uptake) in elite maize cultivars, breeding programs aimed at improving maize have reached a plateau with N recovery efficiency (biomass produced in relation to N applied), which indicates the need to explore opportunities for additional N uptake for enhancing overall NUE (Moose and Below, 2009).

In sorghum however, the N pool does not determine the NUE until about mid-grain filling, with the late grain-filling stage mainly relying on the direct N uptake from soil (McHenry, 2016). Thus, identifying key rooting traits, which can allow for effective N uptake during the mid-to-late-grain-filling phase, could be a promising approach to enhance NUE in sorghum (Nguyen and Kant, 2018). When flooded rice paddies were compared with dry-seeded rice, an advanced and rapid rate of leaf senescence was recorded under dry-seeded rice. This translated to inadequate root length and root tip numbers during the grain maturing phase, which resulted in a deficient supply of N (Hongyan et al., 2018).

**FIGURE 3:**
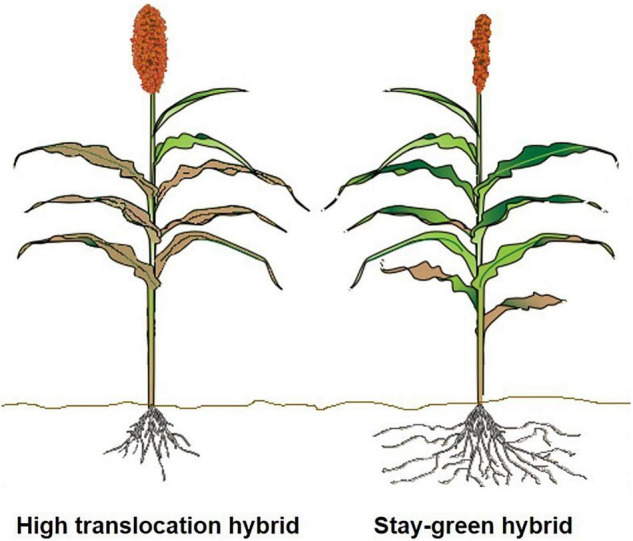
Illustration comparing sorghum hybrids with increased terminal senescence under favorable environmental conditions with greater N translocation from leaves to increase yield and grain quality (left) versus stay-green sorghum hybrids grown under resource-poor conditions (right). Sorghum hybrids with efficient translocation of N and increased senescence under less stressful environments would potentially not require an extensive root system (left).

In sorghum, this could be approached in two different ways. First, sorghum grown under harsh and resource-poor conditions will benefit from the functional stay-green trait, because the impact of the terminal aboveground senescence may be not as severe as detected in other crops such as rice. The active and extended photosynthetic activity due to the stay green trait would possibly continue to support root growth even during late grain filling to actively extend uptake of water and N, as documented by McHenry (2016). We recommend large-scale phenotyping of diversity panels and mapping populations for late-season root characteristics to determine genetic donors that have vigorous rooting systems, which allow for increased late-season uptake of N to support grain filling. For developing proxies for sorghum using recent advances from other plants, based on above-ground foliage to determine below-ground activity, include the following: carbon stocks (Lopatin et al., 2019) and root:shoot ratio in coastal marsh (O’Connell et al., 2015), wheat (Elhaissoufi et al., 2020), and flag-leaf senescence in wheat (Nehe et al., 2018). These studies could provide novel routes for developing sorghum hybrids with increased N uptake to enhance overall NUE. Second, as proposed above, efforts to develop sorghum hybrids with efficient translocation of N could potentially not require an extensively active rooting system, as depicted in Figure 3. To date, there has not been an attempt to understand, under similar management and environmental conditions, the advantage of stay-green hybrids, along with their root systems, under favorable and water-deficit conditions. There can be a potential negative trade-off from stay-greenness that inhibits translocation of N and other nutrients under less stressful or favorable conditions.

Wild or exotic genotypes have the potential to be useful in understanding the pathways leading to enhanced NUE (Garnett et al., 2015). A tremendous amount of genetic diversity within sorghum wild types, related to NUE, has been uncovered, which needs further investigation for translating the potential into adapted varieties (Massel et al., 2016). Massel et al. (2016) reported that a reduction of diversity in 80% of their studied genes in sorghum has been bottlenecked due to domestication. However, the asparagine synthetase (AS), asparaginase (ASPG), and aspartate ammonia lyase (AAL) gene families are all high in sequence diversity, and the (AAL) gene family has the greatest nucleotide diversity, indicting untapped potential. Bollam et al. (2021) screened 60 diverse sorghum genotypes from around the world for NUE and identified candidate lines for enhanced NUE under limited N supply. Genotypes for increased grain, stover, and dual-purpose sorghums were identified. In addition, lines with differing N uptake, assimilation, and amino-acid-biosynthesis genes were identified for incorporation into breeding programs for enhanced sorghum NUE (Bollam et al., 2021). Hence, the identification of diverse landraces or wild accessions could help to increase sorghum’s NUE quickly, particularly because there is a fast turn-around in breeding programs that utilize marker-assisted selection.

Although long-term breeding programs for yield enhancement under optimal N supply have resulted in poor adaptation to low N supply (Garnett et al., 2015), studies in wheat (Hirel et al., 2007) and maize (Moose and Below, 2009) have shown that modern varieties outperform older varieties even under low N-supply environments. Chardon et al. (2010) reported that N uptake and remobilization are independently inherited traits in *Arabidopsis*, which means that favorable alleles could be combined while breeding for enhanced NUE (Xu et al., 2012). Other candidate genes that influence enzymes, including nitrite reductase (NiR), aspartate aminotransferase (AST), and glutamine synthetase (GS) have been identified for further research in breeding for the enhancement of NUE in cereal crops, including sorghum (Massel et al., 2016). These enzymes are key in influencing NUE of plants. In particular, NiR converts NO_3_^–^ into NH_4_^+^, GS influences the assimilation of NH_4_^+^, and AST integrates glutamate into aspartate, which is a precursor for the biosynthesis of different amino acids (Massel et al., 2016). Maranville and Madhavan (2002) explained that assimilation indices were higher for N-insufficient-tolerant lines than for those of intolerant lines under both N sufficient and insufficient environments. They demonstrated that tolerant lines showed higher phosphoenolpyruvate carboxylase activity under N-stress, which suggested that PEPcase and other enzymes that are associated with the synthesis of PEP may be responsible for maintaining higher amounts of photosynthesis under N-stress. Gelli et al. (2014) studied gene-expression patterns between N-insufficient tolerant and intolerant lines and found that there are a number of genes that are indirectly involved in nitrate assimilation. Moreover, differentially expressed gene transcripts (DEG) have been identified, which help to explain how different genotypes react to differing available-N levels at the seedling stage. These DEGs could potentially be candidates for enhancing NUE in sorghum. Xu et al. (2012) suggested that breeding for enhanced NUE should be conducted under moderate N supply to capture the evolutionary trade-offs between high production and the adaption to low N supply. In addition, direct gene transfer with marker-assisted selection approaches should be studied.

Overall, breeding for enhancing NUE in sorghum is difficult, because of the complexity of the trait. But genomic regions and candidate genes have been identified for further investigation. Breeding and improved agronomic practices will have to be carried out simultaneously, aided with accurate phenotyping, to increase NUE of sorghum. A universal method of phenotyping for NUE among all crops should be at the forefront of NUE research to support breeding programs across the world and to establish communication and to translate progress among crops. This would allow for increased genetic gain through enhanced NUE, which would eventually correlate with increased yields and improved quality.

## Future Research Directions

### Optimizing the Stay Green Trait

The relevance of the stay-green trait has been extensively demonstrated under water-limited conditions. But stem sugars and leaf N content locked in the foliage even at maturity (Figure 2) have the potential to increase productivity and grain protein content under favorable conditions. This hypothesis has not been systematically investigated, and, hence, an environment-based testing to determine the degree of stay-greenness needed to sustain productivity, under both water-deficit and favorable conditions, will unravel new opportunities to increase yield and grain quality under a wide range of climatic conditions.

### Post-flowering N Root Uptake

Does sorghum rely on root-based N uptake after flowering that leads to a lush green canopy even at harvest, resulting in poor N translocation? Are the excess sugars in stem and N in leaves locked in foliage an untapped resource that could enhance sorghum productivity and grain quality even under current N management practices? Addressing these questions would help to establish novel experimental outputs, which will allow for enhancement in NUE in sorghum.

### Redesigning the Sorghum Breeding Outlook

Considering sorghum’s ability to thrive under harsh and marginal environments, breeding sorghum for similar or even harsher environments does benefit certain geographic locations. However, sorghum’s genetic yield potential for less stressful environments would need a shift in breeding targets to benefit from traits, such as increased translocation during senescence. Having environment-specific trait(s)-based breeding programs will allow one to utilize sorghum’s inherent ability to tolerate harsh environments, but, at the same time, utilize favorable environments to enhance yield potential.

### High-Throughput Phenotyping for Nitrogen Use Efficiency

Normalized difference vegetation index (NDVI), normalized difference red edge index (NDRE), and other non-destructive, high-throughput sensor technologies to detect nitrogen content in different plant parts, would allow for rapid breeding for increased NUE. High-throughput phenotyping methods and indices using either ground- or aerial-based imaging vehicles will allow for capturing wide genetic diversity for N response and help to integrate NUE traits into routine sorghum breeding programs.

## Conclusion

The importance of NUE in sorghum, is highlighted in this review with the goal of increasing grain yield and quality across a wide range of environments. Enhancing NUE is critical in crops such as sorghum, which is grown predominately on marginal and resource-limited conditions. Though progress on enhancing resilience to harsh environments continues, limited efforts have been invested to explore sorghum’s yield potential under adequate N supply and favorable environments. Grain number is the primary determinant of grain yield in sorghum. Hence adequate availability of N at key physiological stages, e.g., panicle initiation, gametogenesis, and anthesis, is critical to attain maximum yield potential. The stay-green trait, which has been documented to sustain yield under stressed conditions (e.g., drought) could limit the ability to harness yield potential of sorghum, because it retains N and sugars in stem and leaves and prevents them from being translocated to the grain under favorable conditions (adequate moisture and available N). We hypothesize that optimization of the stay-green trait, with a balanced stay-green and senescence ratio, has the potential to increase grain yields and protein levels under favorable environments. To address the need to incorporate large genetic diversity for NUE-related traits, we recommend employing ground- and aerial-based sensor technology and developing new indices to capture these traits. Finally, a better understanding of the genetics and molecular processes involving NUE, accompanied by high-throughput phenotyping, is critical for identifying lines that increase NUE in sorghum.

## Author Contributions

TO and RB drafted the review. MK, SB, and SJ provided comments and edited the manuscript. All authors finalized the review.

## Conflict of Interest

The authors declare that the research was conducted in the absence of any commercial or financial relationships that could be construed as a potential conflict of interest.

## Publisher’s Note

All claims expressed in this article are solely those of the authors and do not necessarily represent those of their affiliated organizations, or those of the publisher, the editors and the reviewers. Any product that may be evaluated in this article, or claim that may be made by its manufacturer, is not guaranteed or endorsed by the publisher.
